# A Systematic Analysis of Research on *Arcobacter*: Public Health Implications from a Food–Environment Interphase Perspective

**DOI:** 10.3390/foods10071673

**Published:** 2021-07-20

**Authors:** Chidozie Declan Iwu, Temitope Cyrus Ekundayo, Anthony Ifeanyin Okoh

**Affiliations:** 1 SAMRC Microbial Water Quality Monitoring Centre, University of Fort Hare, Alice 5700, South Africa; cyruscyrusthem@gmail.com (T.C.E.); AOkoh@ufh.ac.za (A.I.O.); 2 Applied and Environmental Microbiology Research Group, Department of Biochemistry and Microbiology, University of Fort Hare, Alice 5700, South Africa; 3 Department of Biological Sciences, University of Medical Sciences, Ondo PMB 536, Nigeria; 4 Department of Environmental Health Sciences, University of Sharjah, Sharjah P.O. Box 27272, United Arab Emirates

**Keywords:** *Arcobacter*, food safety, public health, environment, gastrointestinal disease, emerging pathogen, enteric bacteria

## Abstract

This review maps the global research landscape of the public health implications of *Arcobacter* from the food–environment interphase using content analytics and integrated science mapping. The search term “Arcobacter” was used to retrieve relevant articles published in Web of Science and Scopus between 1991 to 2019. The number of articles included in the review was 524, with 1304 authors, 172 journal sources, and a collaborative index of 2.55. The annual growth rate of the publications was 9.74%. The most contributing author in the field was Houf K., with 40 publications, 26 h-index, and 2020 total citations. The most productive country was the USA (13.33%). The majority of the articles were published in English (96%) and in the Journal of Food Protection (8.02%). The highest research outputs were in the field of Microbiology (264). The frequently occurred keywords were *Arcobacter*, poultry, shellfish, cattle, and chicken. This study revealed a fair increase in the growth rate of *Arcobacter*-related research—especially in the area of isolation and detection of the pathogen in foods and food environments, as well as the pathogenesis and genetic diversity of the pathogen. Research themes in the area of prevalence and epidemiology seem to be underexplored.

## 1. Introduction

*Arcobacter* is a genus of bacteria that belongs to the Campylobacteraceae family under the *Epsilonproteobacteria* class [1]. They are considered non-spore-forming, spiral-shaped, motile, and fastidious Gram-negative bacteria [2]. In 1991, the genus was proposed to be a group of aerotolerant bacteria [3]. To date, the genus is made up of 27 species possessing substantial genetic diversity with increasing resistance to antibiotics [4].

*Arcobacter* spp. are recently regarded as emerging foodborne zoonotic pathogens affecting both humans and animals. They cause abortion and enteritis in animals and gastroenteritis, diarrhea, and bacteremia in humans [5]. Four species, including *A. butzleri*, *A. cryaerophilus, A. thereius,* and *A. skirrowii*, have been reported to be more clinically involved with human and animal infections [6]. *A. butzleri* has particularly been tagged as a critical threat to human health by the “International Commission on Microbiological Specifications for Foods” [7]. Human infection is usually preceded by the ingestion of contaminated raw or poorly cooked food of animal origin or contaminated water [8].

*Arcobacter* spp. are ubiquitous in the environment and in animals, having a vast range of hosts and habitats [4]. They have been detected in various water sources, such as sewage, lakes, rivers, and plankton [9,10,11], potable water [12,13], domestic and marine water [14], recreational water [15], groundwater [16], and even water delivery pipes [17]. *Arcobacter* spp. have also been found to thrive in the gut and feces of pigs [18,19], poultry meat/carcass [20,21], poultry litter [22], cattle [23], and lamb meat [24]. They have also been identified in dairy products [1,25], shellfish [26,27], and vegetables [28].

*Arcobacter* spp. have shown to be a very important foodborne enteric pathogen, which has captured the interest of numerous researchers worldwide. This has led to the surge in the number of published research articles regarding the pathogen and the nexus of the food environment. For instance, the prevalence of *Arcobacter* in food-processing facilities like poultry [29,30], diary [31,32,33], spinach [34], and beef [35] in countries like Denmark, Belgium, Germany, Malaysia, and Italy have been investigated and published in peer review journals. However, a bibliometric analysis that is required to measure the influence of *Arcobacter*-related publications in the scientific community has not been undertaken. Therefore, we carried out the first bibliometric analysis of *Arcobacter*-related studies between 1991 and 2019 with a specific focus on the food–environment interphase. The findings of this study will help identify the impact of published research articles regarding *Arcobacter* and food–environment interphase. It will also reveal those specific areas that have received increased attention by researchers, the research gaps, and provide evidence for implementing policies that will help curtail the incidence of this pathogen in the food environment, hence minimizing the public health risks posed by the pathogen.

## 2. Materials and Methods

### 2.1. Sources of Arcobacter Research Data

Research data related to *Arcobacter* and its public health implications from the perspective of food–environment interphase between 1991 and 2019 were retrieved from the Web of Science (WoS) and Scopus core database collections. The data were reported according to the preferred reporting items for systematic reviews and meta-analysis (PRISMA) guideline [36]. From the WoS collection, the documents were identified as ‘TITLE: arcobacter*. Timespan: 1991–2019. Indexes: SCI-EXPANDED, SSCI, A&HCI, CPCI-S, CPCI-SSH, BKCI-S, BKCI-SSH, ESCI, CCR-EXPANDED, IC’. From the Scopus collection, the documents were identified as ‘TITLE (arcobacter*) AND PUBYEAR < 2020’.

**Figure 1:**
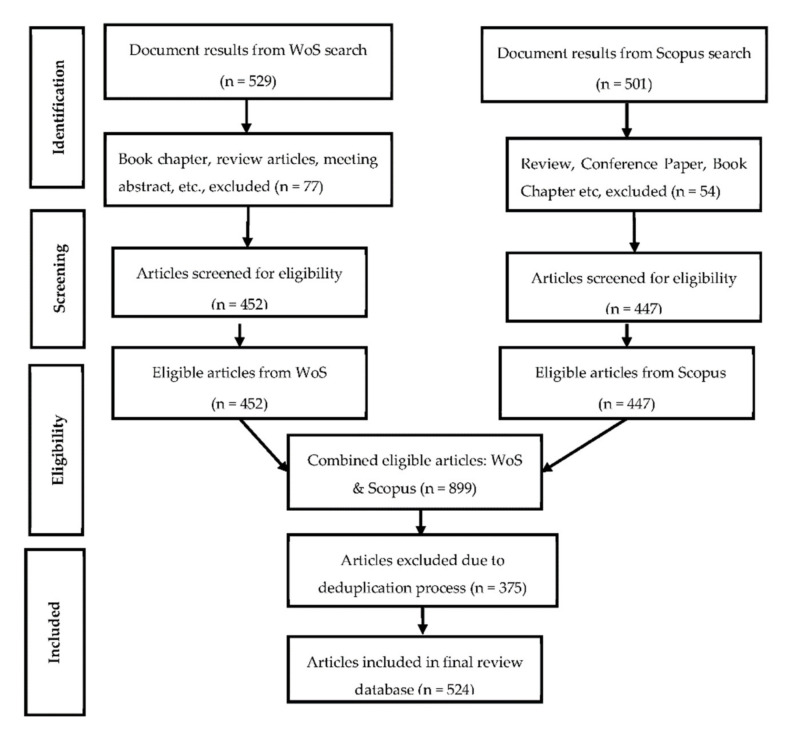
The PRISMA (preferred reporting items for systematic reviews and meta-analysis) flowchart of the bibliometric mapping of research related to *Arcobacter* and its public health implications from the perspective of food–environment interphase.

From the WoS and Scopus databases, 529 and 501 documents were retrieved, respectively, as shown in Figure 1. While 77 documents were excluded from the WoS, 54 documents were excluded from Scopus during the screening process. A total of 899 documents, therefore, met the eligibility criteria, out of which 375 documents were further excluded, due to deduplication, leaving a total number of 524 documents that were finally used in the review.

### 2.2. Bibliometric Analysis of the Data

The data of the bibliometric field were normalized and then analyzed using certain performance indicators like the trend, author rates (in terms of the number of authors, author appearances, authors of single-authored articles, authors of multi-authored articles, single-authored articles, articles/author, authors/article, co-authors/articles), growth analytics and quality metrics (conceptual domain, collaboration index, and H-index), productivity (top productive authors, top institutions, the top countries, total citations per country and top publishing journals), as well as other descriptive indices/rates related to the article, such as annual production and average citations per articles. The conceptual domain and author keywords co-word analysis was factorially mapped using the multiple correspondence analysis as described by [37].

### 2.3. Analysis of the Growth Rate of Arcobacter Research

Analysis of the growth rate of *Arcobacter* research concerning food, environment, microbiology, and public health was carried out. The topical growth analysis, which is based on the average growth rate (AGR) of author-keywords from 2017 to 2019, was done using Equation (1).

(1) $$\mathrm{AGR}=(\overset{2019\mathrm{ey}}{\underset{i=2017\mathrm{sy}}{\sum }}\mathrm{Bi}-\mathrm{Bi}\_1)\;\left(2019\mathrm{ey}-2017\mathrm{sy}\right)+1$$

where AGR: average growth rate., 2017_sy_: start year., 2019_ey_: end year., Bi: number of documents in 2017.

### 2.4. Assessment of Arcobacter Research Scientific Networks

Assessment of *Arcobacter* research scientific networks to determine scientific and intellectual collaborations were done between authors, institutions, or countries using the bipartite vectorial model below:

(2) $$\mathrm{Dw}=D\;\times {\;D}^{T}$$

where *“*Dw is a symmetric matrix (D = D^T^) and composed of author collaboration network (Articles × Authors), country collaboration network (Articles × Countries), and institution collaboration network (Articles × Institutions)”. D is a bipartite network matrix.

The “Fruchterman force-directed algorithms with Jaccard’s similarity index normalization” [38] was used to graph the networks. The nodes/edges in the network indicate the authors, institutions, and countries, while the interconnecting lines indicate the knowledge or resource-sharing relationships. Hierarchical clustering of the authors’ keywords was constructed based on Euclidean distances [39].

### 2.5. Software Analysis

Data were captured in Microsoft Excel (version 2016) and subjected to statistical analysis in R and python program environments using the bibliometrix _R_ package [40], and the ScientoPy python package [41].

## 3. Results and Discussion

### 3.1. The Description of the Bibliometric Data

The information on *Arcobacter*-related research between 1991 and 2019 is shown in Table 1. According to the result, 524 articles from WoS and Scopus published within the date frame, involving 1304 authors, 2662 author appearances, 7 authors of single-authored documents, 1297 authors of multi-authored documents, 15 single-authored documents, and a collaborative index of 2.55 were included in the study. The study revealed 0.402 documents/author, 2.49 authors/document, 5.08 co-authors/document and a collaboration index of 2.55 with 29.23 average citations per documents. It has been shown that the quality of a study in any field is not indicated by the number of citations. Citations rather reflect the scientific impact and readership of the study to other researchers within the field [42], and this is influenced by factors, such as the visibility and accessibility of the article, as well as the year of publication.

**Figure 2:**
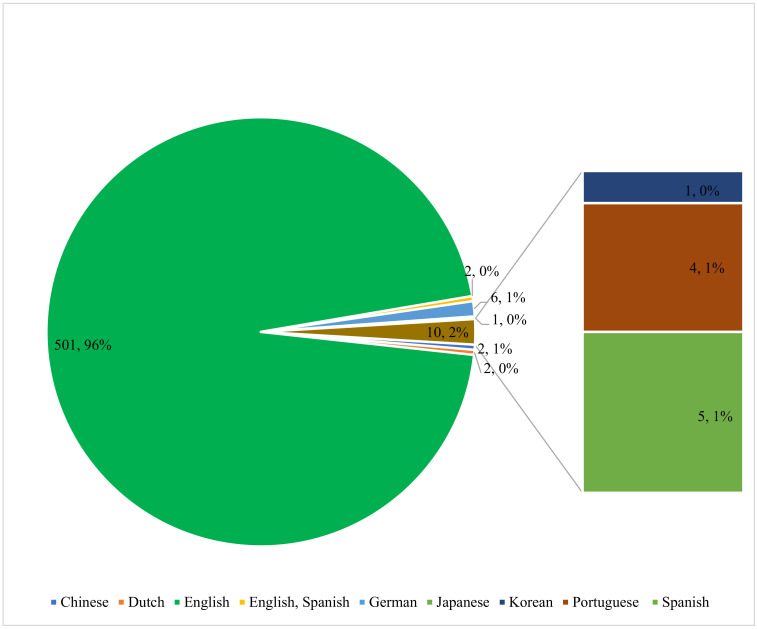
The language diversity of *Arcobacter* publications.

The findings of the study also indicated that the majority of the articles were published in English (n = 501, 96%). Other languages identified follow the order: German (n = 6, 1%), Spanish (n = 5, 1%), Portuguese (n = 4, 1%), Chinese (n = 2, 1%), Dutch (n = 2, 0%), English/Spanish (n = 2, 0%), Korea (n = 1, 0%) and Japanese (n = 1, 0%), as shown in Figure 2. Studies have shown the importance of publishing scientific findings in English. Researchers whose first language is not English are pressured to publish their scientific articles in English [43,44]. While some researchers prefer to use their first non-English languages, especially when the language is used globally, most researchers prefer to use English because of the desire to disseminate their findings to a wider audience, the internationalization of many Universities and research institutes, as well as many scientific journals insisting on the English language [45].

### 3.2. Arcobacter Research Trend

**Figure 3:**
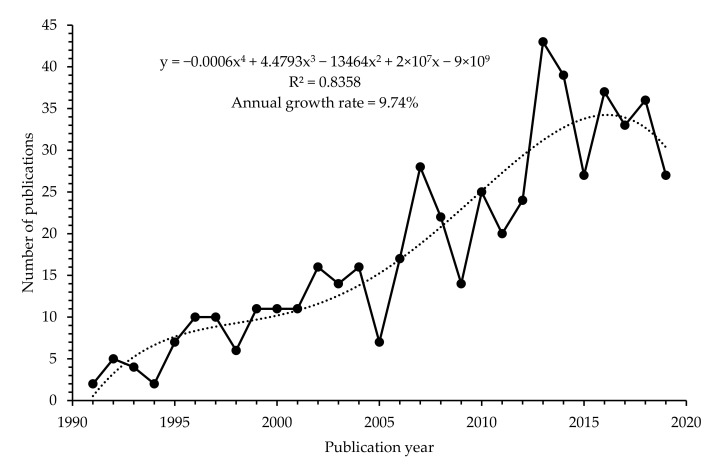
The number of *Arcobacter* publications from 1991 to 2019.

The number of publications between 1991 and 2019 follows a fairly ascending order with an annual growth rate of 9.74% (R^2^ = 0.8358), as shown in Figure 3. Although, a sharp decline in the research outputs in 2005 was observed, which eventually peaked in 2007. This was followed by another sharp decline in 2009, and an increase in 2010. The highest peak of the research outputs was observed in 2013 and has remained unsteady till 2019. The fluctuations in research trends indicate that the publication of *Arcobacter*-related research has not been steady throughout the survey period. This is probably attributed to certain factors, such as available research funds and qualified postgraduate students who can take up *Arcobacter*-related research. Moreover, *Arcobacter* is an emerging pathogen whose evolutionary mechanisms, genetic and ecological diversity are not well understood, hence have not gained much interest from the scientific community. Despite the fluctuations, it is encouraging that there is a steady increase in *Arcobacter*-related research outputs, since the pathogen exhibits significant public health and food safety importance.

### 3.3. Publication Growth

Between 1991 to 2019, the total research outputs according to subject areas ranged from three in Gastroenterology and Hepatology., General and Internal Medicine., Parasitology., and Water Resources to 264 in Microbiology, as shown in Table 2. Also, the average growth rate (AGR) of the research outputs ranged from −0.7 in subject areas like Microbiology., Biotechnology and Applied Microbiology., and Environmental Sciences and Ecology to 1 in Food Science and Technology. Although most research outputs have been in the area of Microbiology, the subject area with the highest average growth rate is in the area of Food Science and Technology. This indicates that *Arcobacter* is an important emerging foodborne pathogen and is often isolated between the food–environment interphase.

The average documents per year (ADY) ranged from 0 in Chemistry., Public, Env. and Occupational Health., Toxicology., Environmental Sciences and Ecology., Research and Experimental Medicine., and General and Internal Medicine to 18.3 in Microbiology, as shown in Table 2. In the same vein, the percentage document per last three years (PDLY) ranged from 0 in Chemistry., Public, Env. and Occupational Health., Toxicology., Environmental Sciences and Ecology., Research and Experimental Medicine., and General and Internal Medicine to 33.3 in Pharmacology and Pharmacy., Gastroenterology and Hepatology., Parasitology., and Water Resources. The highest h-index was observed in subject areas like Microbiology (54)., Biotechnology and Applied Microbiology (40)., Food Science and Technology (32)., Infectious Diseases (21)., Veterinary Sciences (18)., and Immunology (12), as shown in Table 2. This indicates that the productivity and citation impact of *Arcobacter*-related research are within these subject areas. This is not surprising as *Arcobacter* represents a significant infectious disease pathogen with interesting immunological and food safety dynamics.

The most frequently occurred keywords were *Arcobacter*, poultry, shellfish, cattle, and chicken, while the least occurred keywords were vegetables and rivers, as shown in Table S1. This finding is not surprising as most studies on Arcobacter have been on poultry, chicken, and pork [46]. Moreover, poultry serves as an important reservoir of *Arcobacter* spp. and acts as a significant source of spread [47,48]. Although some studies have identified *Arcobacter* spp. in water and vegetables [46], our study demonstrated that there are fewer interests within these two niches based on the generated keywords. Interestingly, the highest average growth rate of *Arcobacter*-related research based on the generated keywords was shellfish with an AGR of 0.7, as shown in Table S1. This indicates an increasing interest in *Arcobacter*-related research with a particular focus on shellfish.

### 3.4. Contributing Authors and Participating Countries in Arcobacter Research

In the present study, Houf K. was identified as the most contributing author, with 40 publications, 26 h-index, 2020 total citations (TC), 7.7 percentage document per last three years (PDLY) of 2000. Other highly productive authors are Figueras M., Vandamme P., Wesley I., Atabay H., Miller W., Giacometti F., etc., as shown in Table 3. However, the contributing author with the highest citation was “Vandamme et al. 1991” with a total citation of 526 and a total citation per year of 18.14, as shown in Table 4. The contributing author with the least citation was “Miller et al. 2007” with a total citation of 133 and a total citation per year of 10.23. A lot of factors play a role in the citation of authors. First is the accessibility of the papers by other researchers in the field. It has been shown that papers that are published in open-access journals are more accessible to other researchers, which consequently leads to their increased citation [49]. Also, published papers in the last 10 to 15 years in open access journals tend to have more citations than those published recently. It has also been shown that some authors are in the habit of citing themselves [50]. This practice can cause a false citation impact on the authors.

Based on the institutional address of the corresponding authors, 36 countries were identified, as shown in Table S2. At least, 10 countries with the highest number of publications are high-income countries. The frequency of publication ranged from 0.23% in China, Mexico, Norway, Poland, Singapore, Switzerland, Thailand to 13.33% in the USA. The AGR ranged from −1.30 in Germany and Canada to 1.30 in Japan. Although *Arcobacter* spp. poses a global challenge, they are more reported in countries like Belgium, the United States of America, Denmark, Brazil, Australia, Italy, the Netherlands, Malaysia, Japan, Spain, Czech Republic, Korea, Egypt, and India, which are mostly high income or middle-income countries [4]. Moreover, it has been shown that high-income countries have a larger pool of trained scientific researchers, more research funding, and more equipped research facilities to isolate and characterize this emerging pathogen [51]. Interestingly, the country with the highest citation is Belgium (T citation = 3737., A citation = 86.91), while the country with the least citation is Singapore (T citation = 0., A citation = 0).

### 3.5. Publication Journals

The 524 papers retrieved in this study were published across 20 most relevant journals, shown in Table S3. The five highest number of publications (n = 42, 8.02%) between the survey period was in the Journal of Food Protection, followed by International Journal of Food Microbiology (n = 25, 4.77%), Journal of Clinical Microbiology (n = 18, 3.44%), Applied and Environmental Microbiology (n = 17, 3.24%) and International Journal of Systematic and Evolutionary Microbiology (n = 17, 3.24%). Elsevier BV/Ltd, the Netherlands, and the American Society for Microbiology, the United States were the most active publishers of *Arcobacter* research with three articles each. The h-index and the total citations of the journals range from 2 and 13 (Italian Journal of Food Safety) to 22 and 1265 (Journal of Food Protection), respectively. The majority of the journals are food, microbiology, and environment-related. The rest are interdisciplinary and have a common scope like tracking or detection of foodborne borne pathogens from food products or the environment.

### 3.6. Collaborations Done in Arcobacter Related Research

**Figure 4:**
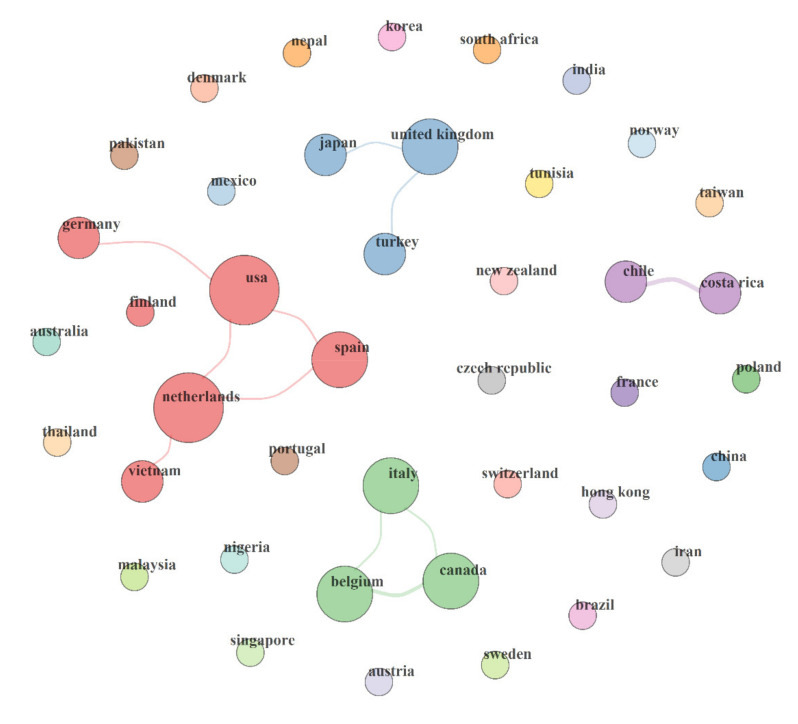
Country’s collaboration map.

Resource- and intellectual sharing forms the basis for scientific networking and collaborations. The country’s collaboration map indicates only four collaboration networks among all the countries involved in *Arcobacter*-related research, as shown in Figure 4. The first network was between Germany, the USA, Spain, and the Netherlands., the second network was between Japan, United Kingdom, and Turkey, the third network was between Italy, Belgium, and Canada., and the fourth network was between Chile and Costa Rica. Interestingly, these collaborations were basically between first-world countries. It has been shown that collaborations between developing and developed countries are rare in several scientific areas [52]. Collaborations between developed and developing countries should be encouraged to increase the exchange of knowledge and ideas relative to each region. With easy communication channels and travel opportunities, the degree of internationalization and exchange of ideas is rising swiftly. This provides an opportunity for universities across the world to form global partnerships and foster relationships with other institutions in other countries.

**Figure 5:**
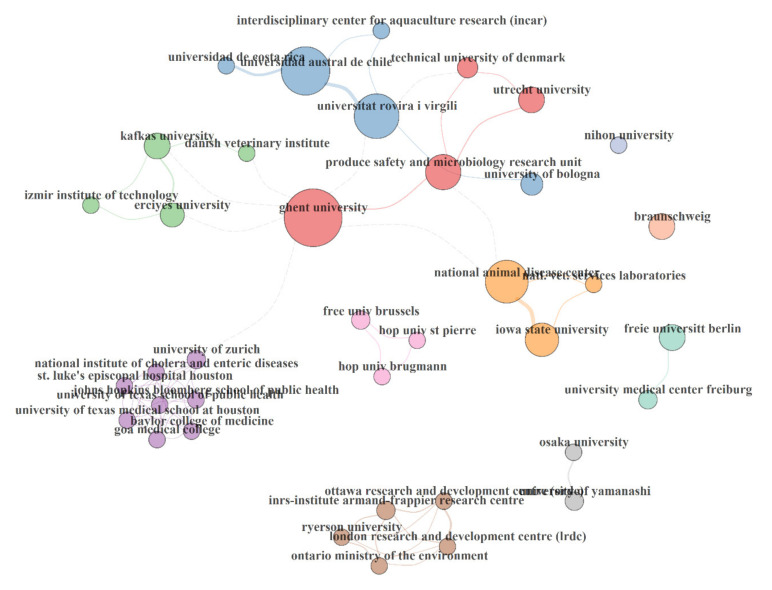
Institutions’ collaboration map.

The present study identified nine institutional collaboration networks involved in *Arcobacter*-related research, as shown in Figure 5. These institutions vary from ministries to research institutes and Universities. Such collaborations have contributed to the progress of science. For instance, researchers from Edinburgh University collaborated with researchers from Harvard University, Peking University, Johns Hopkins University, and Nossal Institute for Global Health at Melbourne University to map out the leading causes of infant mortality in China and how it can be prevented [53,54]. Additionally, institutional partnerships provide opportunities for students and staff members to diversify their research, increase their cultural awareness and have international experiences [53].

**Figure 6:**
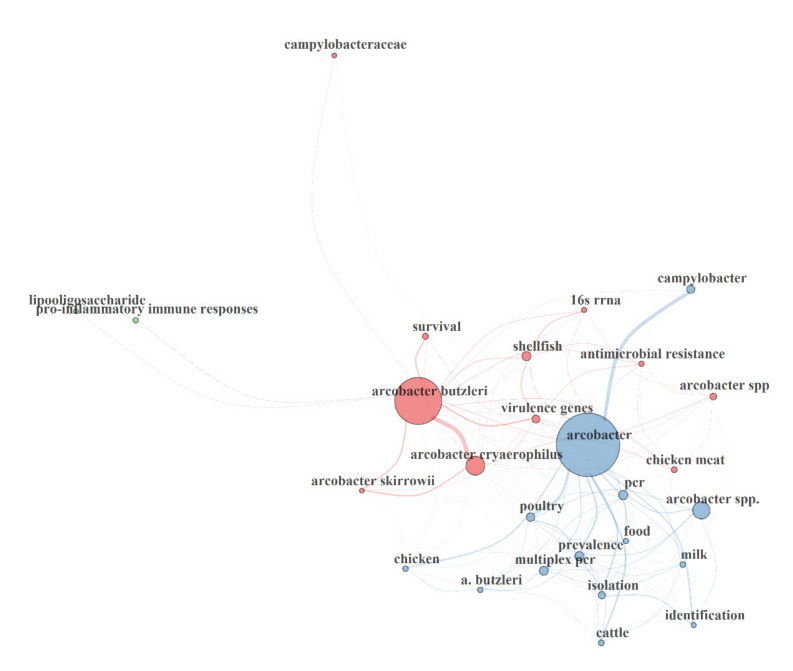
Keywords co-occurrence collaboration map.

**Figure 7:**
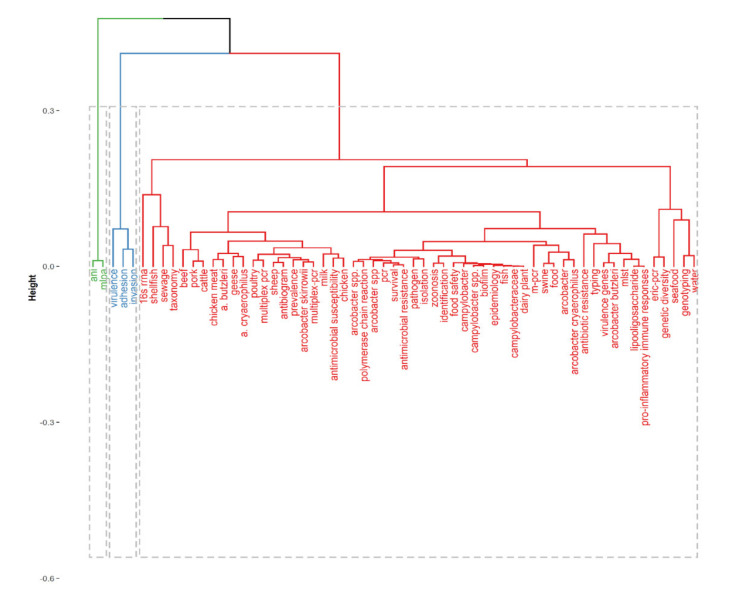
Hierarchical clustering of authors’ keywords.

The keywords co-occurrence collaboration map depicts the hotspots and maps the trends of *Arcobacter*-related research, while the authors’ keyword clusters groups the keywords that have a high correlation with each other [55]. Three keyword collaboration networks (Figure 6) and three keyword clusters (Figure 7), were identified in the present study. The first network (red color) has about 10 nodes (high-frequency words) whose sizes vary depending on the frequency of occurrence of the words. They include “*Arcobacter butzleri*”, “*Arcobacter cryaerophilus*”, “shellfish”, “virulence genes”, “chicken meat”, “antimicrobial resistance”, “survival”, “*Arcobacter* spp.”, “16S rRNA”, “chicken meat” and “*Arcbacter skirrowii*”. Words that frequently appear together in the same source are connected by red lines. Looking at these keywords, it is indicated that hotspots of *Arcobacter*-related research are within these areas and are ranked first in the hierarchical clustering of authors’ keywords. In the same vein, the second network (blue color) has about 14 nodes whose sizes vary depending on the frequency of occurrence of the words. Words like “*Arcobacter*”, “*Arcobacter* spp.”, “Multiplex PCR”, “poultry”, “PCR”, “prevalence” had the largest size of nodes, hence are the frequently occurred words in this network. These keywords indicate an increasing area of research interest and are ranked second in the hierarchical clustering of the authors’ keywords. The third network has only two nodes (green color) whose sizes are very small, indicating a low frequency of occurrence and lack of research interest, thus ranked third in the hierarchical clustering of the authors’ keywords.

### 3.7. Conceptual Frameworks in Arcobacter Research

**Figure 8:**
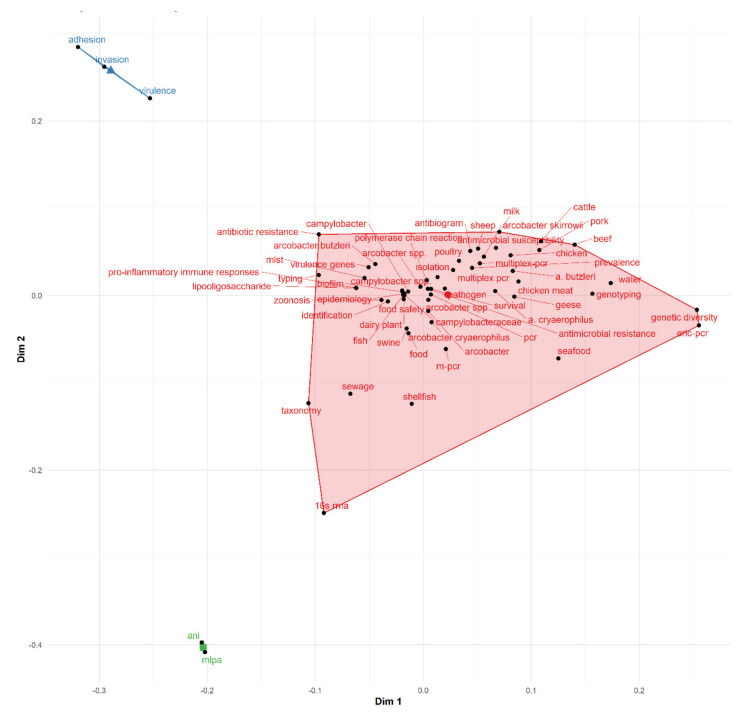
Conceptual frameworks in *Arcobacter* research. Dim 1 = Dimension 1; Dim 2 = Dimension 2.

A conceptual framework depicts what is expected to be found in research. In the present study, three polygons representing three major themes of *Arcobacter*-related research based on authors’ keywords were presented in the common conceptual frames via the k-means clustering, as shown in Figure 8. Based on the generated keywords, the following themes which represents the area where *Arcobacter*-related research is greatly focused on include: (i) The detection and isolation of *Arcobacter* spp in animals, foods, and food environment using molecular methods, which are presented in the red polygon., (ii) Pathogenesis of *Arcobacter* spp. presented in the blue polygon., and (iii) genetic diversity of *Arcobacter* presented in the green polygon. Since *Arcobacter* spp. have been identified as an emerging foodborne pathogen with public health significance, research within these themes seems to be balanced. However, more research in the area of antimicrobial resistance and the epidemiology of the diseases caused by this pathogen should be encouraged.

### 3.8. Description of Studies Published in 2020

Data on *Arcobacter* research outputs published in 2020 are presented in Table S4. From the WoS database, 27 journal articles written in English were retrieved. The majority of the studies were in the area of Biotechnology and Applied Microbiology; Food Science and Technology. Other prominent research areas include Gastroenterology and Hepatology; Microbiology, Food Science and Technology, as well as Biochemistry and Molecular Biology; Chemistry. Other research areas include Medical Laboratory Technology; Infectious Diseases; Agriculture and Engineering; Environmental Sciences and Ecology; and Water Resources. The Applied and Environmental Microbiology journal was the most frequently occurred journal source, followed by the Journal of Food Protection and Food Microbiology. Fanelli et al., 2020 (title: Phenotype and genomic background of *Arcobacter butzleri* strains and taxogenomic assessment of the species) had the highest Cited Reference Count (CRC) of 139, while On et al., 2020 (title: An emended description of *Arcobacter anaerophilus Sasi Jyothsna* et al. 2013: genomic and phenotypic insights) had the lowest CRC of 10.

## 4. Conclusions

The bibliometric analysis on the public health implications of *Arcobacter* from the food–environment interphase between 1991 and 2019 revealed a fair growth rate of publications within the subject area. The majority of the publications were in the field of Microbiology, while the field with the highest average growth rate is Food Science and Technology. There were greater research outputs from high-income countries with little or no collaborations with authors and institutions from low- and middle-income countries. While the research focus and theme of *Arcobacter*-related research are in the detection, and isolation of *Arcobacter* spp. in animals, foods, and food environment using molecular methods, as well as understanding the pathogenesis and genetic diversity of *Arcobacter* spp., areas, such as the epidemiology of this pathogen is underexplored.

## 5. Study Strength and Limitations

This study was the first to provide a comprehensive and global overview of research trends on *Arcobacter* and its public health implications from the perspective of food–environment interphase. However, only studies from the WoS and Scopus databases were considered, thus excluding articles from other databases. Also, articles written in a language other than English were excluded. Furthermore, the quality of studies included in the studies was not assessed.

## Figures and Tables

**Table 1:** Main information about *Arcobacter* data.

Information	Counts/Rates
Documents	524
Sources (Journals, Books, etc.)	172
Keywords Plus (ID)	2778
Authors’ Keywords (DE)	671
Average citations per documents	29.23
Authors	1304
Author Appearances	2662
Authors of single-authored documents	7
Authors of multi-authored documents	1297
Single-authored documents	15
Documents per Author	0.402
Authors per Document	2.49
Co-Authors per Documents	5.08
Collaboration Index	2.55
Document types	
Article	512
Article, book chapter	9
Article, proceedings paper	3

**Table 2:** The Average Growth Rate (AGR) and Average Documents per Year (ADY) of *Arcobacter* research according to subject areas.

Position	Subject	Total	AGR	ADY	PDLY	h-Index
1	Microbiology	264	−0.7	18.3	20.8	54
2	Food Science and Technology	132	1	7.3	16.7	32
3	Biotechnology and Applied Microbiology	128	−0.7	5.7	13.3	40
4	Veterinary Sciences	51	−1	3	17.6	18
5	Infectious Diseases	32	−0.3	1.7	15.6	21
6	Agriculture	18	−0.3	0.7	11.1	9
7	Immunology	16	0	0.7	12.5	12
8	Biochemistry and Molecular Biology	11	−0.3	0.7	18.2	7
9	Science and Technology–Other Topics	10	−1	0.7	20	7
10	Pharmacology and Pharmacy	9	−0.3	1	33.3	6
11	Chemistry	8	0	0	0	4
12	Public, Env. and Occupational Health	6	−0.3	0	0	5
13	Toxicology	6	0	0	0	3
14	Environmental Sciences and Ecology	5	−0.7	0	0	5
15	Research and Experimental Medicine	5	−0.3	0	0	5
16	Gastroenterology and Hepatology	3	0	0.3	33.3	2
17	General and Internal Medicine	3	0	0	0	3
18	Parasitology	3	−0.3	0.3	33.3	3
19	Water Resources	3	0	0.3	33.3	2

PDLY: Percentage document per last three years.

**Table 3:** Most productive authors in *Arcobacter* research.

Position	Authors	PUB	h-Index	TC	PDLY	PY_Start
1	Houf K.	40	26	2020	7.7	2000
2	Figueras M.	32	19	1280	25	2008
3	Vandamme P.	32	25	2646	8.7	1992
4	Wesley I.	26	19	1184	0	1995
5	Atabay H.	23	14	760	0	1997
6	Miller W.	22	8	361	65	2007
7	Giacometti F.	19	9	198	25	2013
8	Serraino A.	19	10	218	18.8	2013
9	Collado L.	18	16	1063	6.2	2008
10	De Z. L.	18	15	1075	0	2000
11	On S.	16	14	1041	15.4	1995
12	Alter T.	14	7	136	7.1	2013
14	Fernndez H.	14	7	114	16.7	1995
15	Levican A.	14	13	590	0	2011
16	Van H. J.	13	13	1050	0	2000
17	Yee E.	13	4	72	91.7	2009
18	Aydin F.	10	7	266	12.5	2001
19	Murano E.	10	9	388	0	1996

PUB: Publications, TC: Total citations, PDLY: Percentage document per last three years, PY_start: Publication start year.

**Table 4:** Top articles per citation.

Rank	Author	Title	TC	TC/Year
1	Vandamme et al., 1991	Proposal for a New Family, Campylobacteraceae	526	18.14
2	Vandamme et al., 1992	Polyphasic Taxonomic Study of the Emended Genus *Arcobacter* with *Arcobacter butzleri* comb. nov. and *Arcobacter skirrowii* sp. nov., an Aerotolerant Bacterium Isolated from Veterinary Specimens	319	11.39
3	Engberg et al., 2000	Prevalence of *Campylobacter*, *Arcobacter*, *Helicobacter*, and *Sutterella* spp. in Human Fecal Samples as Estimated by a Reevaluation of Isolation Methods for Campylobacters	257	12.85
4	Collado et al., 2011	Taxonomy, Epidemiology, and Clinical Relevance of the Genus *Arcobacter*	241	26.78
5	Wirsen et al., 2002	Characterization of an Autotrophic Sulfide-Oxidizing Marine *Arcobacter* sp. That Produces Filamentous Sulfur	225	12.5
6	Houf et al., 2000	Development of a multiplex PCR assay for the simultaneous detection and identification of *Arcobacter butzleri*, *Arcobacter cryaerophilus*, and *Arcobacter skirrowii*	209	10.45
7	Vandenberg et al., 2004	*Arcobacter* Species in Humans	201	12.56
8	Wesley et al., 2000	Fecal Shedding of *Campylobacter* and *Arcobacter* spp. in Dairy Cattle	184	9.2
9	Vandamme et al., 1992	Outbreak of recurrent abdominal cramps associated with *Arcobacter butzleri* in an Italian school.	159	5.68
10	Miller et al., 2007	The Complete Genome Sequence and Analysis of the Epsilonproteobacterium *Arcobacter butzleri*	133	10.23

IF: impact factor; TC: total citation, TC/Year = total citation per year.

## Supplementary Materials

The following are available online at https://www.mdpi.com/article/10.3390/foods10071673/s1, Table S1: Growth of *Arcobacter* research across some selected foods, food products, food animals and environment, Table S2: Country’s contribution to *Arcobacter* research between 1991–2019, Table S3: Most relevant sources (top 20) of studies on *Arcobacter* between 1991–2019, Table S4: *Arcobacter* research data, 2020.
