# Supplementary material for: A Systematic Analysis of Research on Arcobacter: Public Health Implications from a Food–Environment Interphase Perspective
Source: Foods. 2021 Jul 20;10(7):1673. doi: 10.3390/foods10071673 (PMC8303614; doi:10.3390/foods10071673)
Supplement: Supplementary file 1 [file foods-10-01673-s001.zip › foods-1234493-supplementary.pdf]

Table S1. Growth of *Arcobacter* research across some selected foods, food products, food animals and environment.

Position	Author Keywords	Words	Total	AGR	ADY	PDLY	h-index
1	*Arcobacter**	*A. cryaerophilus; A. butzleri; A.; A.* spp.*; A. skirrowii; A. Acticola; A. diarrhea; A. lekithochrous; A. haliotis; A. lacus; A. caeni; A. lekithochrous sp nov; A. suis; A. butzler; A.* spp*; A. selective agars; A. detection; A. faecis; A. nitrofigilis; A. mytili; A.* spp. *detection; A. species; A. identification; A.s; A. sp.; A. butzleri ED-1;A. halophilus*	303	−1.3	25	24.8	42
2	poultry*	poultry meat; poultry; poultry skin; Poultry intestines; poultry processing	21	0.3	1	14.3	10
3	shellfish*	Shellfish	13	0.7	2.7	61.5	8
4	cattle	Cattle; Cow milk	10	−0.3	0.3	10	7
5	chicken	broiler chickens; chicken	10	−0.3	0	0	7
6	milk*	Milk; milk filter	8	0	0.3	12.5	6
7	water*	water; water source; Waterborne pathogen; waterborne pathogen detection	8	−0.3	0.7	25	6
8	beef*	Beef	6	0	0	0	5
9	dog*	Dogs; dog	6	−0.7	0.7	33.3	2
10	pork*	pork abortion; pork chops; Pork	6	0.3	0.7	33.3	4
11	sheep*	Sheep	5	0	0.7	40	3
12	fish*	Fish	4	0	0.3	25	3
13	wastewater*	wastewater; Wastewater treatment	4	0	0.7	50	3
14	geese	Geese	3	0	0.3	33.3	2
15	goat*	Goats	3	0	0.3	33.3	2
16	meat*	Meat	3	0	0	0	3
17	vegetable*	Vegetables; Vegetable processing	2	0	0.3	50	2
18	river*	river water	1	0.3	0.3	100	1
	**Both Keywords**	**Words**	**Total**	**AGR**	**ADY**	**PDLY**	**h-index**
1	Arcobacter*	*A. cryaerophilus; A. butzleri; A.; A.* spp.*; A. skirrowii; A. acticola; A. diarrhea; A. lekithochrous; A. haliotis; A. lacus; A. caeni; A. lekithochrous sp nov; A. molluscorum; A. pacificus; A. porcinus; A. thereius; A. suis; A. butzler; A. spp; A. selective agars; A. detection; A. faecis; A. nitrofigilis; A. mytili; A. spp. detection; A. species; A. identification; A.s; A. sp.; A. butzleri ED-1; A. halophilus*	357	−2.3	27.3	23	45

2	poultry*	Poultry Products; Poultry; poultry meat; poultry skin; Poultry intestines; poultry processing	95	0.3	4	12.6	30
3	water*	Water; Water Treatment-Plant; Waterborne Outbreak; Water Treatment; Water Samples; Water Source; Water Pollution; Water Temperature; Waterborne Pathogen; Waters; Water Supply; Water Microbiology; Water-Quality; Waterborne Pathogen Detection; Water Contamination	75	−1	6	24	26
4	chicken	Broiler-Chickens;Broiler Chickens;Broiler Carcasses;Broiler Flocks;Chicken	61	−0.7	0.7	3.3	28
5	meat*	Meats; Meat	25	0	1.3	16	11
6	shellfish*	Shellfish	21	0.3	3.7	52.4	10
7	cattle	Cattle; Cow Milk	20	0	0.7	10	11
8	milk*	Milk; milk filter	13	0	1	23.1	8
9	beef*	Beef; Beef-Cattle; Beef Trimmings	10	0	0	0	7
10	pork*	pork abortion; pork; pork chops	8	0.3	1	37.5	5
11	dog*	Dogs; dog	6	−0.7	0.7	33.3	2
12	fish*	Fish	5	0	0.3	20	4
13	sheep*	Sheep	5	0	0.7	40	3
14	wastewater*	wastewater; Wastewater treatment	4	0	0.7	50	3
15	geese	Geese	3	0	0.3	33.3	2
16	goat*	Goats	3	0	0.3	33.3	2
17	river*	River Don; River Water	2	0.3	0.3	50	1
18	vegetable*	Vegetables; Vegetable processing	2	0	0.3	50	2

AGR: Average Growth Rate, ADY: Average Documents per Year, PDLY: Percentage document per last 3 years.

Table S2. Country's contribution to *Arcobacter* research between 1991–2019.

	Corresponding Author's Countries									Total Citations per Country		
	**Country**	**PUB**	**Freq (%)**	**AGR**	**PDLY**	**SCA**	**SCA/PUB (%)**	**MCA**	**MCA/PUB (%)**	**Country**	**TC**	**AAC**
1	USA	58	13.33	1.00	26	53.00	91.38	5	8.62	Belgium	3737	86.91
2	Belgium	43	9.89	−0.30	10.6	41.00	95.35	2	4.65	USA	2580	44.48
3	Spain	40	9.20	−0.70	26.8	40.00	100.00	0	0	Spain	1566	39.15
4	Italy	38	8.74	0.30	26.2	38.00	100.00	0	0	United Kingdom	1180	35.76
5	United Kingdom	33	7.59	0.00	0	31.00	93.94	2	6.06	Italy	757	19.92
6	Germany	29	6.67	−1.30	15.2	29.00	100.00	0	0	Denmark	591	65.67
7	Chile	24	5.52	−0.70	15	22.00	91.67	2	8.33	Canada	479	31.93
8	Czech Republic	22	5.06	0.30	22.7	22.00	100.00	0	0	Germany	396	13.66
9	Turkey	21	4.83	−1.00	17.6	21.00	100.00	0	0	Turkey	346	16.48
10	Canada	15	3.45	−1.30	13	14.00	93.33	1	6.67	Chile	314	13.08
11	India	11	2.53	−1.00	27.8	11.00	100.00	0	0	Japan	269	26.9
12	Japan	10	2.30	1.30	50	10.00	100.00	0	0	Netherlands	240	24
13	Netherlands	10	2.30	0.30	7.7	7.00	70.00	3	30	France	178	59.33
14	Brazil	9	2.07	0.70	55.6	9.00	100.00	0	0	Czech Republic	166	7.55
15	Denmark	9	2.07	0.30	5.6	9.00	100.00	0	0	Austria	161	53.67
16	Korea	9	2.07	0.00	12.5	9.00	100.00	0	0	Korea	155	17.22
17	Portugal	8	1.84	0.30	44.4	8.00	100.00	0	0	India	146	13.27
18	Costa Rica	6	1.38	−0.70	30	4.00	66.67	2	33.33	Portugal	142	17.75
19	Malaysia	6	1.38	0.00	33.3	6.00	100.00	0	0	Taiwan	141	70.5
20	Iran	4	0.92	−0.30	14.3	4.00	100.00	0	0	Hong Kong	136	68
21	Australia	3	0.69	0.70	28.6	3.00	100.00	0	0	Australia	126	42
22	Austria	3	0.69	0.00	0	3.00	100.00	0	0	Malaysia	93	15.5
23	France	3	0.69	0.00	0	3.00	100.00	0	0	South Africa	88	44
24	New Zealand	3	0.69	0.30	25	3.00	100.00	0	0	New Zealand	80	26.67
25	Nigeria	3	0.69	0.00	16.7	3.00	100.00	0	0	Iran	67	16.75
26	Finland	2	0.46	0.00	0	2.00	100.00	0	0	Costa Rica	58	9.67
27	Hong Kong	2	0.46	0.00	0	2.00	100.00	0	0	Brazil	55	6.11

28	South Africa	2	0.46	0.00	0	2.00	100.00	0	0	Mexico	45	45
29	Taiwan	2	0.46	0.00	0	2.00	100.00	0	0	Nigeria	25	8.33
30	China	1	0.23	0.00	0	1.00	100.00	0	0	Finland	24	12
31	Mexico	1	0.23	0.00	0	1.00	100.00	0	0	Norway	24	24
32	Norway	1	0.23	0.00	0	1	100.00	0	0	Poland	17	17
33	Poland	1	0.23	0.00	0	1	100.00	0	0	Switzerland	8	8
34	Singapore	1	0.23	0.00	0	1	100.00	0	0	Thailand	5	5
35	Switzerland	1	0.23	0.00	0	1	100.00	0	0	China	1	1
36	Thailand	1	0.23	0.00	0	1	100.00	0	0	Singapore	0	0

PUB: publications, Freq: frequency, TC: total citations, AGR: Average growth rate, PDLY: percentage document per last 3 years, SCA: Single Country Article, MCA: Multiple Country Article, TC: Total citations, AAC: Average Article citations.

Table S3. Most relevant sources (top 20) of studies on *Arcobacter* between 1991–2019.

Rank	Sources	PUB	% Freq	h_index	TC	SJR	Country	PU	PDLY	PY_start
1	Journal Of Food Protection	42	8.02	22	1265	0.61	United States	International Association for Food Protection	9.8	1996
2	International Journal Of Food Microbiology	25	4.77	18	1037	1.38	Netherlands	Elsevier BV	12	1996
3	Journal Of Clinical Microbiology	18	3.44	17	1306	2.31	United States	American Society for Microbiology	0	1992
4	Applied And Environmental Microbiology	17	3.24	15	1083	1.66	United States	American Society for Microbiology	5.9	2000
5	International Journal Of Systematic And Evolutionary Microbiology	17	3.24	13	709	0.91	United Kingdom	Society for General Microbiology	37.5	2005
6	Journal Of Applied Microbiology	17	3.24	12	651	0.83	United Kingdom	Blackwell Publishing Inc.	17.6	1997
7	Letters In Applied Microbiology	16	3.05	13	695	0.57	United Kingdom	Blackwell Publishing Inc.	5.9	1996
8	Archiv Fur Lebensmittelhygiene	11	2.1	4	35				0	2006
9	Microbiology Resource Announcements	11	2.1	3	16				100	2018
10	Veterinary Microbiology	11	2.1	10	449	1.17	Netherlands	Elsevier BV	0	1997
11	Food Microbiology	10	1.91	7	134	1.4	United States	Elsevier Inc.	30	2001
12	Foodborne Pathogens And Disease	10	1.91	6	106	0.83	United States	Mary Ann Liebert Inc.	30	2010
13	Frontiers In Microbiology	10	1.91	4	91	1.63	Switzerland	Frontiers Media S.A	77.8	2014
14	Plos One	9	1.72	5	216	1.1	United States	Public Library of Science	12.5	2007
15	Systematic And Applied Microbiology	9	1.72	9	406	1.3	Netherlands	Elsevier BV	0	1995
16	Folia Microbiologica	8	1.53	5	75	0.5	Netherlands	Springer Netherlands	28.6	2003

17	Genome Announcements	7	1.34	4	38	0.49	United States	American Society for Microbiology	0	2013
18	Brazilian Journal Of Microbiology	6	1.15	6	83	0.74	Brazil	Elsevier Editora Ltda	0	2001
19	Italian Journal Of Food Safety	6	1.15	2	13	0.35	Italy	PagePress	66.7	2013
20	Journal Of Medical Microbiology	6	1.15	5	293	0.87	United Kingdom	Society for General Microbiology	25	2006

PUB: publications, Freq: frequency, TC: total citations, SJR: PU: Publishers, PDLY: percentage document per last 3 years, PY: Publication year.

Table S4. *Arcobacter* research data, 2020.

S/N	Authors	Article Title	Source Title	CRC	TC, WC	180 DUC	Research Areas	Author Keywords
1	Khodamoradi et al., 2020	The incidence and antimicrobial resistance of Arcobacter species in animal and poultry meat samples at slaughterhouses in Iran	Iranian Journal of Microbiology	33	0	0	Microbiology	Arcobacter; Broiler chicks; Iran; Prevalence; Antimicrobial susceptibility
2	Jribi et al., 2020	Occurrence and Antibiotic Resistance of Arcobacter Species Isolates from Poultry in Tunisia	Journal of Food Protection	68	0	3	Biotechnology & Applied Microbiology; Food Science & Technology	Antimicrobial susceptibility; Arcobacter butzleri; Arcobacter cryaerophilus; Arcobacter spp; Matrix-assisted laser desorption ionization-time of flight mass spectrometry
3	Silha et al., 2020	Chemical Composition of Natural Hydrolates and Their Antimicrobial Activity on Arcobacter-Like Cells in Comparison with Other Microorganisms	Molecules	77	0	1	Biochemistry & Molecular Biology; Chemistry	Lavandula angustifolia; Syzygium aromaticum; Foeniculum vulgare; Laurus nobilis; hydrolates; distillation; Arcobacter-like bacteria; antimicrobial activity; biofilm formation; gas chromatography
4	Mottola et al., 2020	Arcobacter species detection in Italian composite foods	LWT–Food Science and Technology	25	0	2	Food Science & Technology	Ready-to-eat composite food; Mozzarella roll; Arcobacter butzleri; Virulence factors
5	Vidal-Veuthey et al., 2020	Antimicrobial resistance and virulence genes profiles of Arcobacter butzleri strains isolated from back yard chickens and retail poultry meat in Chile	Letters in Applied Microbiology	35	0	4	Biotechnology & Applied Microbiology; Microbiology	Arcobacter; Chile; resistance; Valdivia; virulence

6	Celik et al., 2020	Isolation of Arcobacter spp. and identification of isolates by multiplex PCR from various domestic poultry and wild avian species	Annals of Microbiology	49	0	3	Biotechnology & Applied Microbiology; Microbiology	Arcobacter spp.; Cloacal swab; feces; Poultry; Wild bird
7	Niedermeyer et al., 2020	Search for Campylobacter spp. Reveals High Prevalence and Pronounced Genetic Diversity of Arcobacter butzleri in Floodwater Samples Associated with Hurricane Florence in North Carolina, USA	Applied and Environmental Microbiology	49	0	2	Biotechnology & Applied Microbiology; Microbiology	Arcobacter; Arcobacter butzleri; Campylobacter; Campylobacter jejuni; floodwaters; hurricane; MLST; genotype
8	Wang, KD et al., 2020	Arcobacter Identification and Species Determination Using Raman Spectroscopy Combined with Neural Networks	Applied and Environmental Microbiology	72	0	30	Biotechnology & Applied Microbiology; Microbiology	Arcobacter; Raman spectroscopy; rapid identification; machine learning; convolutional neural network; fully connected artificial neural network
9	Rahman et al., 2020	Improved culture enrichment broth for isolation of Arcobacter-like species from the marine environment	Scientific Reports	76	0	4	Science & Technology–Other Topics	
10	On et al., 2020	A critical rebuttal of the proposed division of the genus Arcobacter into six genera using comparative genomic, phylogenetic, and phenotypic criteria	Systematic and Applied Microbiology	44	1	1	Biotechnology & Applied Microbiology; Microbiology	Arcobacter; Polyphasic taxonomy; Phylogenomics; Comparative genomics; Reclassification; Rebuttal
11	Szydlowski et al., 2020	Metabolic engineering of a novel strain of electrogenic bacterium Arcobacter butzleri to create a platform for single analyte detection using a microbial fuel cell	Enzyme and Microbial Technology	65	0	2	Biotechnology & Applied Microbiology	Biosensor; CRISPR; Electrode associated bacteria; Electron transfer; Microbial fuel cell

12	Fanelli et al., 2020	Phenotype and genomic background of Arcobacter butzleri strains and taxogenomic assessment of the species	Food Microbiology	139	4	4	Biotechnology & Applied Microbiology; Food Science & Technology; Microbiology	Arcobacter butzleri; Aliarcobacter butzleri; Lithotrophic bacteria; Vegetables; Shellfish; Antibiotic and heavy metal resistance
13	Marta et al., 2020	Large genetic diversity of Arcobacter butzleri isolated from raw milk in Southern Italy	Food Microbiology	68	2	0	Biotechnology & Applied Microbiology; Food Science & Technology; Microbiology	Arcobacter; Genotyping; Multi locus sequence typing (MLST); Real-time PCR; Bulk tank milk
14	Salinas et al., 2020	Arcobacter butzleri, a zoonotic agent in bovine milk	Acta Bioquimica Clinica Latinoamericana	15	0	0	Medical Laboratory Technology	Arcobacter; Zoonosis; Milk
15	Isidro et al., 2020	Virulence and antibiotic resistance plasticity of Arcobacter butzleri: Insights on the genomic diversity of an emerging human pathogen	Infection Genetics and Evolution	82	5	1	Infectious Diseases	Arcobacter butzleri; Genome diversity; Virulence factors; Antibiotic resistance; porA; Phase variation
16	Simaluiza et al., 2020	Prevalence of zoonotic Arcobacter species in pigs at slaughterhouse level in Ecuador	Revista Mvz Cordoba	19	0	0	Agriculture	Antimicrobial drug resistance; epidemiology; reservoir; zoonoses (Source: DeCS)
17	Kristensen et al., 2020	Bacteria from the Genus Arcobacter Are Abundant in Effluent from Wastewater Treatment Plants	Applied and Environmental Microbiology	64	3	6	Biotechnology & Applied Microbiology; Microbiology	Arcobacter; wastewater; activated sludge; full-scale wastewater treatment plants; removal efficiency
18	Nelapati et al., 2020	Occurrence, virulence gene and antimicrobial susceptibility profiles of Arcobacter sp. isolated from catla (Catla catla) in India	Letters in Applied Microbiology	40	0	1	Biotechnology & Applied Microbiology; Microbiology	antibiogram profile; Arcobacter; freshwater fish; virulence; MDR

19	Bruckner et al., 2020	Prevalence and antimicrobial susceptibility of Arcobacter species in human stool samples derived from out- and inpatients: the prospective German Arcobacter prevalence study Arcopath	Gut Pathogens	41	0	0	Gastroenterology & Hepatology; Microbiology	Arcobacter; Humans; Germany; Prevalence; Antimicrobial susceptibility
20	Shange et al., 2020	Prevalence of Campylobacter and Arcobacter Species in Ostriches from Oudtshoorn, South Africa	Journal of Food Protection	41	0	0	Biotechnology & Applied Microbiology; Food Science & Technology	Arcobacter; Campylobacter; Ostriches; Poultry; Prevalence; South Africa
21	Sharma et al., 2020	Molecular detection and genetic characterization of Arcobacter butzleri isolated from red-footed pet tortoises suspected for Campylobacter spp. from Grenada, West Indies	Plos One	70	0	0	Science & Technology–Other Topics	
22	Carney et al., 2020	Highly heterogeneous temporal dynamics in the abundance and diversity of the emerging pathogens Arcobacter at an urban beach	Water Research	74	1	4	Engineering; Environmental Sciences & Ecology; Water Resources	Arcobacter; Stormwater; Pathogens; Oligotyping; Microbial ecology
23	Aydin et al., 2020	Prevalence of Arcobacter and Campylobacter in beef meat samples and characterization of the recovered isolates	Journal of Consumer Protection and Food Safety	57	1	3	Food Science & Technology	Antibacterial susceptibility; Arcobacter spp; Beef meat; Campylobacter spp; ERIC-PCR
24	Cruzado-Bravo et al., 2020	Occurrence of Arcobacter spp. in Brazilian Minas frescal cheese and raw cow milk and its association with microbiological and physicochemical parameters	Food Control	51	2	1	Food Science & Technology	Emerging pathogen; Microbiological and physicochemical analysis; Somatic cell counts; Milk components
25	Miller et al., 2020	Complete Genome Sequencing of Four Arcobacter Species Reveals a Diverse Suite of Mobile Elements	Genome Biology and Evolution	31	0	0	Evolutionary Biology; Genetics & Heredity	Arcobacter; insertion sequences; mobile elements; horizontal gene transfer

26	Bruckner et al., 2020	Characterization of Arcobacter strains isolated from human stool samples: results from the prospective German prevalence study Arcopath	Gut Pathogens	41	3	0	Gastroenterology & Hepatology; Microbiology	Arcobacter; Human; Cytotoxicity; Virulence genes; Genotyping
27	On et al., 2020	An emended description of Arcobacter anaerophilus Sasi Jyothsna et al. 2013: genomic and phenotypic insights	International Journal of Systematic and Evolutionary Microbiology	10	1	0	Microbiology	Arcobacter; Arcobacter anaerophilus; revised taxonomic description; Halarcobacter anaerophilus

CRC: Cited Reference Count, DUC: Day Usage Count, TC: Times Cited, WC: WoS Core.
